# Caregiver burden among informal caregivers in the largest specialized palliative care unit in Malaysia: a cross sectional study

**DOI:** 10.1186/s12904-020-00691-1

**Published:** 2020-12-08

**Authors:** Zati Sabrina Ahmad Zubaidi, Farnaza Ariffin, Cindy Teoh Cy Oun, Diana Katiman

**Affiliations:** 1 Clinical lecturer and Family Medicine Specialist, Department of Primary Care Medicine, Faculty of Medicine, Universiti Teknologi MARA (UiTM) Selayang campus, Jalan Prima 7, 68100 Batu Caves, Selangor Malaysia; 2 Palliative Medicine Physician, Palliative Care Unit, Selayang Hospital, 68100 Batu Caves, Selangor Malaysia; 3 Clinical lecturer and Palliative Medicine Physician, Department of Medicine, Faculty of Medicine, Universiti Teknologi MARA (UiTM) Sg Buloh campus, Jalan Hospital, 47000 Sungai Buloh, Selangor Malaysia

**Keywords:** Caregiver, Burden, Palliative

## Abstract

**Background:**

Informal caregivers (IC) are often overshadowed by the attention required by the terminally ill. This study aims to reveal the estimated proportion of caregiver burden, psychological manifestations and factors associated with caregiver burden among IC in the largest specialized Palliative Care Unit (PCU) in Malaysia.

**Methods:**

This was a cross-sectional study involving IC attending a PCU. Caregiver burden and psychological manifestations were measured using previously translated and validated Zarit Burden Interview and DASS-21 questionnaires respectively. Two hundred forty-nine samples were selected for analysis.

**Result:**

The mean ZBI score was 23.33 ± 13.7. About half of the population 118(47.4%) was found to experienced caregiver burden whereby majority have mild to moderate burden 90(36.1%). The most common psychological manifestation among IC is anxiety 74(29.7%) followed by depression 51(20.4%) and stress 46(18.5%). Multiple logistic regression demonstrated that women who are IC to patients with non-malignancy were less likely to experience caregiver burden. IC who were highly educated and spent more than 14 h per day caregiving were at least twice likely to experience caregiver burden. Finally, those with symptoms of depression and anxiety were three times more likely to suffer from caregiver burden.

**Conclusion:**

Caregiver burden among IC to palliative patients is prevalent in this population. IC who are men, educated, caregiving for patients with malignancy, long hours of caregiving and have symptoms of depression and anxiety are at risk of developing caregiver burden. Targeted screening should be implemented and IC well-being should be given more emphasis in local policies.

## Background

In 2017, the life expectancy at birth in Malaysia is 72.7 years for male and 77.4 years for female [1]. Over the years, longer life expectancy coupled with declining fertility rates has led to an increase in the elderly and young adult ratio [2]. Therefore, Malaysia is projected to witness an exponential increment in the demand for palliative care and the number of informal caregivers (IC) [3]. Informal caregiver is defined as the individual responsible for the patient care at home and carrying out specific instructions given by their treating health care personnel [4].

Palliative care in Malaysia has been around for nearly 30 years. However, the delivery of the services is still limited. Domiciliary care in public primary care clinics are only available in four out of 13 states and is still in the fledgling stage. Therefore, community palliative care is largely provided by charitable non-governmental organizations. At present there are only five specialized palliative care units (PCU) in Malaysia and many of its expertise are congregated in urban areas. It is projected that by 2030, 239,713 Malaysians will require palliative care [3]. However, only 10% of palliative care needs are being met in the country [3].

Realistically, it will take many years for the Malaysian palliative care services to grow to its optimal standard. Inadequate financial support undermines infrastructural development efforts and the current economic atmosphere will need time to regenerate itself. Considerable amount of time is also required to train qualified palliative physicians. Therefore, the health care system relies heavily on IC to support the care for palliative patients especially in rural parts of the country. However, similarly to many developing countries, there is no regulated system in place that identifies IC concerns, needs, gaps and barriers.

In Malaysia, filial obligation is a cultural norm. For many years, family members are socially assigned, morally obliged and intrinsically assumed to care for the unwell family member [5, 6]. In Malaysia, the tradition of family caregiver is fundamental in the community setting whereby a significant proportion of elderlies live together and are taken care of by their children especially when the spouse has passed on [7]. Nevertheless, previous local research reported that family members have increasingly finding it challenging to care for their older generations [8]. Over the past several decade, Malaysia has witnessed massive urban migration, especially inter-urban migration and rural-urban migration as young adults search for better job opportunities [9]. However, inflation and increasing cost of living in the city coupled with slow economic growth have resulted in urban poverty [8, 9]. Financial constraints may become barriers to caregiving. Additionally, despite preventive strategies, cancer patients are often presented late, resulting in many caregivers finding themselves unprepared to shoulder on the responsibilities [10]. Culturally, resentment over caregiving may lead to family disputes and being labelled as ungrateful to the sacrifice made by their elders [6]. Hence, IC do not voluntarily express their suffering without careful probing [6]. Therefore, caregiver burden among IC may be under reported.

Caregiver burden is defined as the strain borne by a person who cares for a chronically ill, disabled or elderly person [11]. It is also the caregiver’s personal perceptions about the extent of caregiving having an impact on their emotional, social, financial, physical and spiritual functioning [12]. Caregiver burden is highly subjective [4]. However, the subjective burden is a key mediator in the framework that relates to the demands of caregiving and caregiving outcome, notably, caregiver burnout [4]. Caregiver burnout or compassion fatigue is a condition where IC experience a state of physical, emotional and mental exhaustion that can alter their attitude towards the patient from caring and nurturing to becoming completely unconcerned [13]. At this state of mind, IC will no longer be reliable to attend to the patient’s basic needs. This may lead to neglect and abuse [14]. Previous literature review have demonstrated that caregiver burden is significantly associated with being female, having low education, staying together with the patient, spending long hours caregiving, having underlying depression, living in social isolation and having financial constraints [15].

Apart from the impact it has on patients, the consequences of caregiver burden has many adverse effect on caregivers. IC have reported deteriorating physical health, psychological complications and poor job performance [16, 17]. The most common physical complaints include sleep disturbance, prolong fatigue, debilitating pain, loss of physical strength, loss of appetite, and weight loss [18]. Caregiver burden has also been associated with immune dysregulations, coronary artery disease and even an increased in all-cause mortality [19, 20]. IC often becomes the “invisible patient” as they commonly have needs that are overshadowed by the complexity and urgency of the patient’s care. As a result, IC are exposed to psychological complications such as depression, anxiety and stress [21].

In contrary, recent findings have demonstrated that caregiving for cancer and dementia patients have been associated with post traumatic growth and improvement in family resilience [22]. Over time, family members facing persistent challenges from caregiving to these conditions were found to develop personal and relational transformation to grow out of adversity. Post traumatic growth and family resilience have been associated with a reduction in the risk of depression, anxiety and stress [22]. The positive outcome can also be explained by the subjective nature of caregiver burden where IC may suffer from burden but value the experience as it contributed to their own self-enrichment [22]. Considering the contradictory findings of the effect of caregiving and the unique Malaysian population where filial obligation is a learned tradition, research on the association between caregiver burden among palliative patients, their psychological manifestation and other potential risk factor in this population is warranted.

Despite the growing interest in palliative care research, there has been very few analytical studies on the predictors of caregiver burden to IC among palliative care patients in Malaysia. Therefore, the objectives of this study are to (i) determine the proportion of IC experiencing burden and its severity, (ii) determine the psychological manifestations (depression, anxiety and stress) from caregiving and (iii) determine the factors associated with caregiver burden among IC to palliative patients in the largest specialized Palliative Care Unit (PCU) in Malaysia.

## Methods

### Study design and setting

This was a cross-sectional study conducted at Malaysia’s largest specialized PCU in a government tertiary hospital located in the state of Selangor, Malaysia. The center was chosen for this study because of its public accessibility with a considerable patient load and good representation of multiracial diversity. The PCU consists of an in-patient ward and an outpatient clinic.

### Study population

The inclusion criteria for this study were (i) being the primary caregiver. (The primary caregivers were individuals who the patient felt most involved in the caregiving process), (ii) caregivers who are ≥18 years old and (iii) able to understand English or Malay language. The exclusion criteria were (i) caregivers who were mentally challenge which is associated with loss of a sense of reality (schizophrenia, bipolar disorder, acute psychosis or dementia), (ii) appears to be in emotional distress and (iii) caregivers to patients who are institutionalized.

### Sampling method

The caregivers were each approached individually at different times in PCU using a non-probability, convenience sampling. They were given explanations and a participant information sheet regarding the study. Those who expressed interest to participate were screened for the inclusion and exclusion criteria and were asked to give written informed consent.

### Study tools

The instrument used for this study consisted of three parts. The first part captures the caregivers sociodemographic (age, gender, ethnicity, having a religion/faith, education level, employment status, marital status and income status), the care-receiver characteristics (age, gender and main diagnosis), patient functional status measured by the Eastern Cooperative Oncology Group (ECOG) performance scale and attributes of caregiving (having other caregiving responsibility, relationship with patient, having underlying medical illness, staying together with patient, being paid and total hours of caregiving. The total hours of caregiving are categorized into more than 14 h per day or less [7]. The second part is the Zarit Burden Interview (ZBI) which consists of 22-items. The ZBI reflects on issues pertaining to health, finances, social life, emotional well-being, personal life, and interpersonal relationship. The self-administered questionnaire is scored using a 5-point Likert scale that ranges from 0 (never) to 4 (nearly always). The minimum score is 0 and the maximum score is 88. Respondents can be classified into having caregiver burden (22–88) and no burden (≤ 21). Respondents suffering from caregiver burden can further be classified into mild to moderate (22–40), moderate to severe (41–60) and severe burden (61–88). The ZBI is the oldest and the most utilized instrument to assess caregiver burden in clinical and research settings [23]. The Malay version of ZBI have demonstrated a good psychometric property with a Cronbach Alpha of 0.89 [23]. The third part is the Depression, Anxiety and Stress Scales (DASS-21) questionnaire. The Malay version of DASS-21 has been validated extensively in the local population. The Cronbach’s alpha values are 0.75, 0.74 and 0.79 respectively for depression, anxiety and stress sub scales [24]. DASS-21 has 21 questions which uses a 4-point Likert scale that ranges from 0 (did not apply at all) to 3 (applies to me most of the time). It consists of three subscales which measures depression (7 items), anxiety (7 items) and stress (7 items). For each subscale, the sum of the score is multiplied by two. Therefore, the minimum score is 0 and the maximum score is 42 whereby they could be further categorized into being normal, mild, moderate, severe and extremely severe symptoms. It is, however, not a tool to diagnose clinical depression and anxiety. The instrument was chosen because it is the most widely used tool to screen for symptoms of depression, anxiety and stress due to its practicality in usage for research purposes and screening at community health settings [24].

### Data collection and procedures

Caregivers were asked to answer the questionnaires separately from the patient if the situation permits to minimize response bias. Data collection was conducted from September until December 2019. Data was collected by a research assistant who was trained with regards to the study procedures to minimize variability in the method of data collection.

### Questionnaire administration

**Fig. 1:**
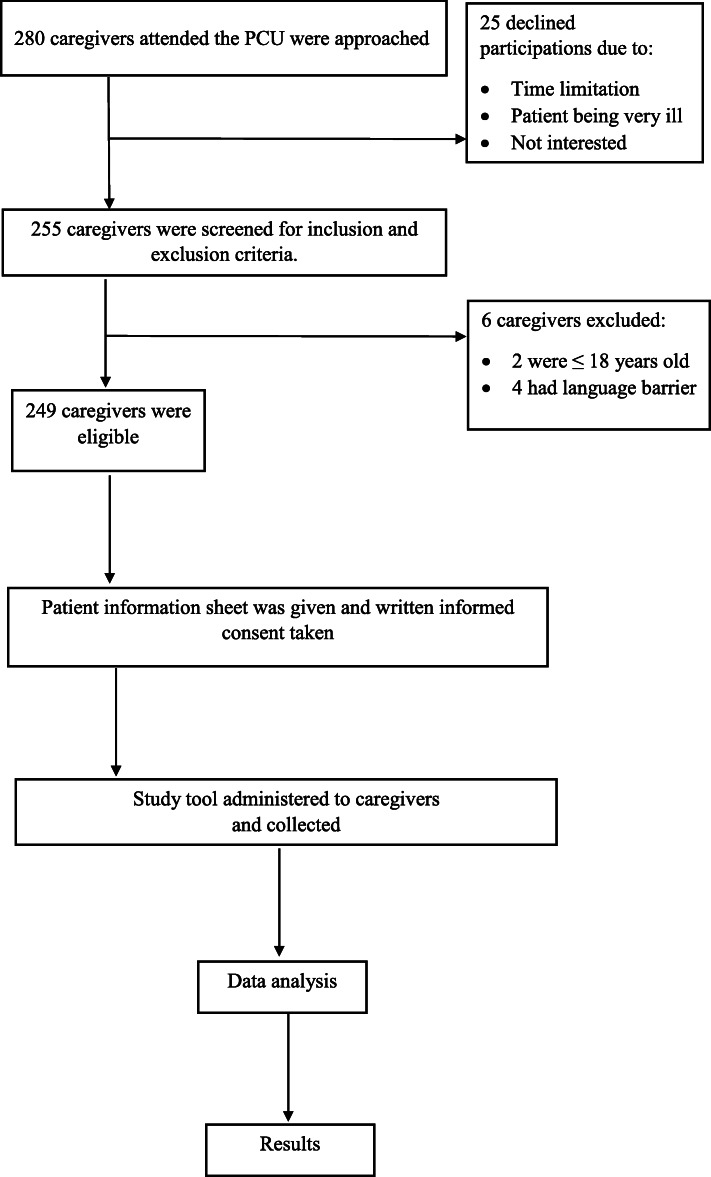
Flow chart of the conduct of the study

Participants who were eligible were approached individually and given verbal instructions to circle or tick the best option that they consider appropriate in the questionnaire. They were advised to ask for clarifications from the investigators should any questions arise and to provide their own individual response. The enumerator ensured that participants were not in a hurry, distracted or interacting with other people during questionnaire administration. Participants took approximately 15 to 20 min to complete the questionnaires. Figure 1 illustrates the conduct of the study.

### Sample size calculation

Sample size calculation was calculated using the OpenEpi sample size calculator that is available online (www.openepi.com) using sample size for proportion formula with 5% precision and 95% confidence interval. The population size (N) was based on the data retrieved from the largest community palliative care provider in Malaysia which is 2076 patients that was referred in a year [25]. The hypothesized % frequency of outcome factor in the population (p) was estimated based on the highest frequency of caregivers among elderly with chronic illness experiencing burden in our local population from previous available recent study which was 21.7% [7]. Therefore, sample size aimed for analysis was 224. Taking into consideration 20% of non-responders, 280 informal caregivers were approached.

### Statistical analysis

Data was analyzed using IBM SPSS Statistics 23. Normality test was verified using the *Kolmogorov-Smirnov* test prior to analysis. Qualitative variables were presented as absolute and relative frequencies while quantitative variables as mean with standard deviation (SD). Cronbach’s alpha was used to verify the reliability of the MZBI in this population, with an internal consistency of 0.89. Some variables were regrouped into meaningful categories for analysis and they are ethnicity, income level, education level, marital status, relationship with patient, patient diagnosis and ECOG functional status. To identify the association between caregiver burden and palliative patients who have become bedridden, The ECOG performance scale is categorize into ECOG < 3 (patients who are not bedridden) and ECOG ≥3 (patients who are bed ridden) [26]. Chi square test and independent t test were used to analyze the association of each variable with the outcome for categorical and continuous variables respectively. Variables with *P* value of less than 0.25 in the univariate analysis were included in the multiple logistic regression using stepwise regression method. Variables with *p* < 0.05 were considered statistically significant. The two-way interaction between each variable were checked and it was found to be insignificant. Multicollinearity was tested using Variance Inflation Factor (VIF). Model fitness was checked using Hosmer Lemeshow goodness of fit test.

## Result

### Sociodemographic characteristics of caregivers

The mean age of caregivers was 48.78 ± 12.99 years. Majority of the caregivers were female (70.3%). About half of them were Malay in ethnicity (51%), followed by Chinese (34.9%) and Indian (11.6%) which demonstrated a good representation of the country’s ethnic distribution. A minority of them (6.8%) did not have any religious belief which is also quite rare in this country. 36.1% were highly educated (received tertiary education up to college or university) and most of them were married (75.1%). Approximately half of them (51%) were employed and caregiving for more than 14 h per day (54.6%). About a third of them had other caregiving responsibilities besides the patient (37.3%) and underlying medical condition (30.9%). Majority of them are either the children (49.4%) or spouse (34.1%) to the patient. Spousal caregivers are mostly the wife to the patient. Most of them are staying together with the patient (80.3%). Table 1 summarizes the results.

**Table 1:** Socio-demographic characteristic of caregivers

Demographic characteristics	Frequency n (%)	Mean ± SD
**Age (years)**		48.78 ± 12.99
**Gender**		
Male	74 (29.7)	
Female	175 (70.3)	
**Ethnicity**		
Malay	127 (51)	
Chinese	87 (34.9)	
Indian	29 (11.6)	
Others	6 (2.4)	
**Having a religion / faith**		
Yes	232 (93.2)	
No	17 (6.8)	
**Highest education level**		
None	3 (1.2)	
Primary school	17 (6.8)	
Secondary school	139 (55.8)	
College/University	90 (36.1)	
**Employment status**		
Employed for wages	91 (36.5)	
Self-employed	36 (14.5)	
Retired	24 (9.6)	
Housewife	51 (20.5)	
Not working	47 (18.9)	
**Marital status**		
Married	187 (75.1)	
Unmarried	51 (20.5)	
Divorced	10 (4)	
Widowed	1 (0.4)	
**Income status**		
Low income	195 (78.3)	
Middle income	47 (18.9)	
High income	7 (2.8)	
**Other caregiving responsibilities**		
Yes	93 (37.3)	
No	156 (62.7)	
**Relationship with patient**		
Spouse	85 (34.1)	
• Husband	25 (10)	
• Wife	60 (24)	
Children	123 (49.4)	
Daughter/son in law	12 (4.8)	
Grandchildren	7 (2.8)	
Maid	5 (2.0)	
Friend	2 (0.8)	
Niece	4 (1.6)	
Parents	7 (2.8)	
Siblings	4 (1.6)	
**Underlying medical illness**		
Yes	77 (30.9)	
No	172 (69.1)	
**Staying together with patient**		
Yes	200 (80.3)	
No	49 (19.7)	
**Paid to take care of this patient**		
Yes	22 (8.8)	
No	227 (91.2)	
**Total hours caregiving per day**		
0–13	113 (45.4)	
14–24	136 (54.6)	

### Patients (care-receivers) characteristics

The mean age of the patients was 67 ± 13.76 years. There was almost an equal distribution between male (50.6%) and female (49.4%) patients. The most common diagnosis is malignancy (73.9%). More than half of the patients have an ECOG performance scale of ≥3 whereby they are mostly confined to bed (55.4%). Table 2 summarizes patients (care-receivers) characteristics.

**Table 2:** Patients (care-receivers) characteristics

Characteristics	Frequency n (%)	Mean ± SD
**Age**		67 ± 13.76
**Gender**		
Male	126 (50.6)	
Female	123 (49.4)	
**Diagnosis**		
Malignancy	184 (73.9)	
End stage renal failure	54 (21.7)	
Cardiovascular diseases	2 (0.8)	
Central Nervous System	3 (1.2)	
Very Elderly	2 (0.8)	
Liver cirrhosis	4 (1.6)	
**ECOG performance scale**		
(0) Fully active, able to carry on all pre-disease performance without restriction.	3 (1.2)	
(1) Restricted in physically strenuous activity but ambulatory and able to carry out work of a light or sedentary nature	32 (12.9)	
(2) Ambulatory and capable of all selfcare but unable to carry out any work activities; up and about more than 50% of waking hours.	76 (30.5)	
(3) Capable of only limited selfcare; confined to bed or chair more than 50% of waking hours	65 (26.1)	
(4) Completely disabled; cannot carry on any selfcare; totally confined to bed or chair	73 (29.3)	

### Caregiver burden among IC to palliative patients

The mean ZBI score among IC was 23.33 ± 13.7. Interestingly, almost half of the population, 118 (47.4%) was found to experienced caregiver burden. Fortunately, majority suffers from mild to moderate burden 90 (36.1%) followed by moderate to severe burden 24 (9.6%) and severe burden 4 (1.6%).

### The psychological manifestation exhibited among caregivers to palliative patients

The most common psychological manifestation among caregivers is anxiety 74 (29.7%) whereby most of them 25 (10%) reported moderate intensity. This is followed by depression 51(20.4%) and stress 46 (18.5%). Table 3 summarizes the results.

**Table 3:** Psychological manifestation exhibited among caregivers

Symptoms	Frequency n (%)
**Depression**	**51 (20.4)**
Mild	20 (8)
Moderate	18 (7.2)
Severe	6 (2.4)
Extremely severe	7 (2.8)
**Anxiety**	**74 (29.7)**
Mild	23 (9.2)
Moderate	25 (10)
Severe	10 (4)
Extremely severe	16 (6.4)
**Stress**	**46 (18.5)**
Mild	17 (6.8)
Moderate	21 (8.4)
Severe	5 (2)
Extremely severe	3 (1.2)

### Association between caregiver burden with sociodemographic of caregivers, caregiving attributes and psychological manifestations (depression, anxiety and stress)

There is a significant association between caregiver burden with being highly educated and presence of psychological manifestation such as depression, anxiety and stress. (*p*< 0.05). Most of IC who were highly educated (57.8%) experienced caregiver burden. 76.5, 71.6 and 73.9% of IC who were suffering from caregiver burden were having symptoms depression, anxiety and stress respectively. Table 4 summarizes the results.

**Table 4:** Association between caregiver burden with sociodemographic of caregivers, caregiving attributes and psychological manifestations (depression, anxiety and stress)

Variables	Burdened (***N*** = 118)		Not Burdened (***N*** = 131)		Mean diff (95% CI) ͣ or x^**2**^ (df)^**b**^	***P*** value
	n (%)	Mean ± SD	n (%)	Mean ± SD		
**Age**		47.64 ±12.24		49.79 ±13.6	−2.15 (−5.39,1.09)^a^	0.193
**Gender**					2.714 (1)^b^	0.099
Male	41 (55.4)		33 (44.6)			
Female	77 (44)		98 (56)			
**Ethnicity**					0.479 (2)^b^	0.787
Malay	61 (48)		66 (52)			
Chinese	39 (44.8)		48 (55.2)			
Indian and others	18 (51.4)		17 (48.6)			
**Having a religion / faith**					0.226 (1)^b^	0.635
Yes	109 (47)		123 (53)			
No	9 (47.4)		8 (52.6)			
**Education level**					6.101 (1)^b^	0.014
Low (Secondary school and below)	66 (41.5)		93 (58.5)			
High (College/University)	52 (57.8)		38 (42.2)			
**Employment status**					3.546 (4)^b^	0.471
Employed for wages	44 (48.4)		47 (51.6)			
Self-employed	20 (55.6)		16 (44.4)			
Retired	13 (54.2)		11 (45.8)			
Housewife	19 (37.3)		32 (62.7)			
Not working	22 (46.8)		25 (53.2)			
**Marital status**					0.033 (1)^b^	0.856
Married	88 (47.1)		99 (52.9)			
Unmarried	30 (48.4)		32 (51.6)			
**Income status**					0.016 (1)^b^	0.900
Low	92 (47.2)		103 (52.8)			
Middle to High	26 (48.1)		28 (51.8)			
**Caregiving for others**					3.304 (1)^b^	0.069
Yes	51 (54.8)		42 (45.2)			
No	67 (42.9)		89 (57.1)			
**Relationship with patient**					1.516 (2)^b^	0.469
Spouse	43 (50.6)		42 (49.4)			
Children	59 (48)		64 (52)			
Others	16 (39)		25 (61)			
**Underlying illness**					1.534 (1)^b^	0.216
Yes	41 (53.2)		36 (46.8)			
No	77 (44.8)		95 (55.2)			
**Staying with patient**					1.057 (1)^b^	0.304
Yes	98 (49)		102 (51)			
No	20 (40.8)		29 (59.2)			
**Being paid**					1.325 (1)^b^	0.250
Yes	13 (59.1)		9 (40.9)			
No	105 (46)		122 (54)			
**Hours of caregiving per day**					2.002 (1)^b^	0.157
< 14	48 (42.5)		65 (57.5)			
> 14	70 (51.5)		66 (48.5)			
**Depression**					21.756 (1)^b^	0.0001
**Yes**	**39 (76.5)**		**12 (23.5)**			
Level						
Mild	11 (55)		9 (45)			
Moderate	16 (88.9)		2 (11.1)			
Severe	5 (83.3)		1 (16.7)			
Extremely severe	7 (100)		0 (0)			
**No**	**79 (39.9)**		**119 (60.1)**			
**Anxiety**					24.798 (1)^b^	0.0001
**Yes**	**53 (71.6)**		**21 (28.4)**			
Level						
Mild	17 (73.9)		6 (26.1)			
Moderate	16 (64)		9 (36)			
Severe	7 (70)		3 (30)			
Extremely severe	13 (81.3)		3 (18.8)			
**No**	**65 (37.1)**		**110 (62.9)**			
**Stress**					15.921 (1)^b^	0.0001
**Yes**	**34 (73.9)**		**12 (26.1)**			
Level						
Mild	12 (70.6)		5 (29.4)			
Moderate	14 (66.7)		7 (33.3)			
Severe	5 (100)		0 (0)			
Extremely severe	3 (100)		0 (0)			
**No**	**84 (41.4)**		**119 (58.6)**			

^a^ Independent t-test

^b^ Chi square test

### Association between caregiver burden with patients (care-receivers) characteristics

Patients with a malignancy diagnosis were found to be significantly associated with caregiver burden (*p*<0.05). Among IC who were experiencing caregiver burden, 52.2% of the care-receivers were diagnosed with a cancer diagnosis compared to 47.8% among those without burden. Table 5 summarizes the results.

**Table 5:** Association between caregiver burden with patients (care-receivers) characteristics

Variables	Burdened		Not Burdened		Mean diff (95%CI) ͣ or x^**2**^ (df)^**b**^	***P*** value
	n (%)	Mean ± SD	n (%)	Mean ± SD		
**Age**		66.7 ±12.15		67.89 ±13.91	−1.724 (−5.17,1.72)^a^	0.325
**Gender**					0.107 (1)^b^	0.743
Male	61 (48.4)		65 (51.6)			
Female	57 (46.3)		66 (53.7)			
**Diagnosis**					6.471 (1)^b^	0.011
Malignancy	96 (52.2)		88 (47.8)			
Non -malignancy	22 (33.8)		43 (66.2)			
**ECOG performance scale**					0.167 (1)^b^	0.682
ECOG < 3	51 (45.9)		60 (54.1)			
ECOG ≥3	67 (48.6)		71 (51.4)			

^a^ Independent t-test

^b^ Chi square test

### Multiple logistic regression on factors associated with caregiver burden among palliative patients

Variables with significance level of *p* < 0.25 in the bivariate analysis which are caregivers to a cancer patient, education level, caregiver’s age, gender, other caregiving responsibilities, underlying medical illness, being paid, hours of caregiving, depression, anxiety and stress were selected into the multiple logistic regression. The preliminary model showed that caregivers’ gender, education level, hours of caregiving, symptoms of depression and anxiety as well as caregiving to a cancer patient were found to be significant associated factors. The final model depicts that caregivers who are female and caregiving for non-cancer patients were less likely to experience caregiver burden. Caregivers who were highly educated and spent more than 14 h per day caregiving were at least twice likely to experience caregiver burden. Finally, caregivers who had symptoms of depression and anxiety were three times more likely to suffer from caregiver burden. Multicollinearity analysis found that all variables have tolerance of more than 0.1 and VIF of less than 5, confirming that there is no problem with multicollinearity that requires remedial measures. Nagelkerke *R*^*2*^ value is 0.298. Hosmer Lemeshow goodness of fit test and was found to be not significant at *p* = 0.713, proving that the model is fit. Table 6 summarizes the results.

**Table 6:** Multiple logistic regression on factors associated with caregiver burden among palliative patients

Variables		Adjusted OR (95% CI)	Wald statistics (df)	***P*** value ª
**Gender**	Female	0.515 (0.268,0.99)	3.957 (1)	0.047
	Male	1.00		
**Education level**	High	2.414 (1.266,4.603)	7.163 (1)	0.007
	Low	1.00		
**Hours of caregiving**	≥ 14 h	2.084 (1.139,3.813)	5.672 (1)	0.017
	< 14 h	1.00		
**Depression**	Yes	3.041 (1.130,8.179)	4.852 (1)	0.028
	No	1.00		
**Anxiety**	Yes	3.299 (1.533,7.099)	9.316 (1)	0.002
	No	1.00		
**Caregiver to a cancer patient**	No	0.422 (0.207,0.862)	5.603 (1)	0.018
	Yes	1.00		

ªMultiple logistic regression. The model reasonably fits well. Model assumptions were met. There was no interaction and multicollinearity problem

## Discussion

### Caregiver burden among IC to palliative patients

It is important to note that about half of IC to palliative patients in this population were experiencing mild to moderate burden as the mean ZBI score was 23.33 ± 13.7. The mean ZBI score in this population is lower compared to previous local studies using similar instrument involving other medical conditions. In 2003, caregiver burden was found to be higher among elderly patients with dementia (35.4 ± 15.8) [27]. However, the services, awareness and support that were available two decades ago in the Malaysian health care setting was not as widespread as today. In a more recent study, caregiver burden among cancer patients were found to be slightly higher than the current population (median 26) [28]. Nevertheless, this study was conducted in the state of Sarawak, an eastern part of Malaysia which has a less developed health infrastructure. In Turkey, caregiver burden was higher among patients with hematological cancer compared to the study population (28.41 ± 13.90) [29]. This might be because the study population was undergoing active intervention whereby peripheral blood transplant was undertaken. The level of caregiver burden was also found to vary depending on the progression of the disease. In South England, caregiver burden to advanced cancer patients was found to be lower compared to this study population (median 18.5). An explanation for this is that the patients recruited in that study were already terminal, whereby caregivers may have either reached acceptance or the contact with the patients were only in a matter of weeks [30]. This is supported by a study in Ontario whereby caregiver burden to terminally ill breast cancer patients were significantly lower compared to the initial phase of palliative intervention. (26.2 and 19.4, *p* = 0.02) [31].

### Factors associated with caregiver burden

#### Psychological manifestations among caregivers

Psychological manifestations are common among caregivers. However, because it is infrequently screened, most of the cases are under diagnosed [32]. Caregivers with symptoms of depression were found to be three times more likely to experience caregiver burden in this population. Most of the caregivers experiencing burden who had symptoms of depression reported moderate intensity. Psychological manifestation among caregivers have a direct effect on the care-receivers. Depression among caregivers have been associated with neglect in many studies [33–35]. This can be explained by the characteristics of depression itself, whereby patients usually experience extreme fatigue and inhibition [36]. Caregivers with symptoms of anxiety were also found to be three times more likely to experience caregiver burden in this population. Most of the caregivers experiencing burden who had symptoms of anxiety reported mild intensity. A study among family caregivers to dependent elderlies demonstrated that caregivers with anxiety were twice as likely to have abusive risk factors towards the care-receivers. Anxiety was translated into experiencing tension which resulted in violent behavior against elderlies and it has been reported to worsen overtime [36]. Lack of caregiver training and education may also predispose to anxiety. Caregivers who are not aware about the signs and symptoms of dying commonly perceive the conditions as suffering, which may lead to feelings of anxiety [37]. A previous study has shown that caregivers who perceived that patients had unmet needs at the end of life reported higher objective burden [38]. Nevertheless, it is important to note that depression and anxiety are not mutually exclusive and that it commonly co-exists.

#### Gender difference in caregiver burden

Interestingly, this study has demonstrated that female caregivers experience lesser burden compared to male. Many previous literatures have shown that female caregivers reported greater burden than male [5, 39]. This is because women in many parts of the world endures unequal distributions of rewards, privileges, opportunities, and responsibilities that forces them into the caregiving role which subsequently hampers their other functioning such as their health and professional career [39]. Women have also reportedly felt more obligated towards caregiving compared to men when given the task and men more often than not received assistance from others [39]. However, among patients with mental illness there has been no significant difference between gender among those experiencing caregiver burden [40]. But among cancer survivors with poor psychosocial functioning, male caregivers have reported greater stress from caregiving [41]. This can be explained by the demographic of the study population whereby most of the women are spousal caregivers. Culturally in most parts of the world, spousal caregiving is an extension of women’s social role which begins soon after they are married [39]. This provides mental preparedness to care for the terminally ill husband. Whereas for men, having to shoulder on the task which is unfamiliar and sudden may create anxiety and stress [39]. Women and men deal with caregiving differently even if the situations are the same. Male caregivers lean towards a managerial approach while female caregivers adapt to a more nurturing, hands-on role that is emotionally focussed [42]. In other words, men prefer to manage care rather than administer care. For example, male caregivers are more likely to hire someone to deliver care and are less comfortable providing personal care such as bathing and dressing compared to women.

#### Caregiving for a cancer patient

Previous research has shown that a malignancy diagnosis is associated with higher caregiver burden [43]. Literatures have describe that the burden experienced by IC to cancer patients is multidimensional whereby the physical, social and economic burden are all intertwined [44–46]. The trajectory of the disease is variable and caregivers experiences varies according to the stage of their involvement. Caregivers to cancer patients who become physically dependent may exhibit higher level of burden, especially among those who have limited social interaction and restricted daily activities from their involvement in the patients daily needs [46]. Previous literature demonstrated that the constant need for assistance in the patients’ daily activity of living among cancer patient has been associated with caregiver burden [47]. The relationship and age of caregivers to cancer patients have also shown to be associated with caregiver burden. Siblings to patients reported higher level of burden compared to other IC [37]. A systematic review has shown that younger caregivers are more predisposed to caregiver burden [47]. Finally, caregivers to patients with solid tumours (especially non-breast related tumours) were found to be more predisposed to experience burden [48].

#### Caregivers education level

Education level has long been associated with health literacy. Caregivers with good education are presumed to be more resourceful and problem-focused during crises. However, higher health literacy could also mean that caregivers are made aware of the prognosis and subsequent challenges that they will encounter. Such knowing may contribute to unwanted psychological manifestation that creates emotional burden. As depicted in this population, IC who are highly educated have double the odds of experiencing caregiver burden. In Germany, similar finding were found whereby educated IC experienced more caregiver burden [49]. In Netherlands, educated caregivers to cancer patients post-discharge were associated with low caregiver’s esteem [50]. Caregivers with low self-esteem have been associated with caregiver burden [51]. Oedekoven M. et al. explained that highly educated caregivers are worried about the loss of self-fulfillment and autonomy when caregiving for the terminally ill [49] This creates fear and builds up the feeling of burden. Caregiving demands among educated caregiver may also interfere with their work performance compared to those who are lesser educated. Most of them hold important positions that require concentration, commitment, bigger responsibilities or longer working hours [52]. Spousal caregivers to the terminally ill have reported negative consequences in their career in a way of diminished productivity, decreased quality of work, and missed opportunities for promotion [53]. A systematic review has also shown that caregivers who are simultaneously working are given less priority in caregiver management [5].

#### Long hours of caregiving

The wear-and-tear hypothesis predicts that the longer caregiving is sustained, the greater the decline in caregivers’ subjective well-being [54]. This study has found that caregivers who spend more than 14 h a day caregiving are twice likely to suffer from caregiver burden. This finding supports another local study that was conducted among IC to elderly with chronic disease whereby those who spend more than 14 h per day providing care were found to be five times more likely to suffer from high burden [7]. Another study which was done among caregivers to patients with Alzheimer’s confirms that, the number of hours caregiving have a direct effect on caregiver burden [55]. Research has also demonstrated that caregivers who are given a break for 3 h or more from their duties were less likely to suffer from severe caregiver burden [56]. However, a study among caregivers to dementia patients found inconsistent results on the association of the duration of caregiving with caregiving outcomes [57]. The long hours of caregiving often include interrupted hours at night. Caregiver burden has been associated with poor sleep quality [58, 59]. Poor sleep quality may precipitate negative affective, behavioural and cognitive responses that include irritability, negative thoughts, decreased concentration and poor motivation which can intensify caregiver burden [59].

### Study limitation

The cross-sectional design of this study is unable to determine the causal relationship between the different variables and caregiver burden. The self-reporting method is vulnerable to social desirability bias, respondent’s honesty, their individual understandings and their introspective ability. Therefore, the findings from this study should be interpreted with caution.

### Clinical implications and suggestions

An important finding from this study is that caregiver burden is prevalent among the Malaysian palliative IC. Therefore, it is crucial to systematically screen IC for risk of burden, psychological complications such as depression and anxiety as well as needs assessment so that support can be targeted to their specific issues. In a busy clinic, “high risk” IC such as men who appear to be educated and caregiving for cancer patients should alert clinicians to offer necessary screening for caregiver burden. IC with evidence of burden should be documented in the electronic medical record for further follow up to prevent poor bereavement outcome and to alert other interdisciplinary clinicians such as psychiatrist or family medicine specialist who may come across this patient for mental health issues. For palliative patients planned from ward discharge, a protected time of transition period should be allocated for the IC a few days prior to discharge to allow for a structured training session among IC while still under supervision. Caregiver education has been associated with improvement in caregiver esteem and confidence which reduces the risk of developing caregiver burden [60].

Long hours of caregiving should be a serious subject of concern. The Malaysian government has shown inclinations in financially assisting IC by allowing yearly income tax redemption for items or medications purchased to care for their parents. A significant relief would be in the form of physical assistance by establishing a partially government subsidized private home care assistance to achieve reasonable hours of caregiving among IC as private home care services can be expensive. An effective caregiver can have a positive economic impact as it allows patients to remain within their home environment, hence reducing the usage of health resources and avoiding multiple admission to the hospitals [22]. Malaysia is also in the need to develop a respite center. To date, there are only a few affordable and established respite facility that is available in the country for palliative patients. As the number of palliative population is expected to grow, this facility will be of great help to provide quality temporary care to allow IC to attend to their errands, work or even go on a short trip to come back rejuvenated.

Theoretically, integrating IC has always been a fundamental feature in palliative care management. However, previous research has suggested that effective strategies in managing IC are lacking and it is generally not delineated clearly. This is because research on interventions and comprehensive guidelines for IC are limited, therefore training in this specific area is superficial [61]. Hence, more local research is needed to convince stakeholders to provide funding allocations, professional training and improve current policy on the management of caregivers.

## Conclusions

Caregiver burden among IC to palliative patients in this population is prevalent. IC who are men, highly educated, caregiving for cancer patients and exposed to long hours of caregiving are at risk of developing caregiver burden. Psychological manifestation such as symptoms of depression and anxiety are evident in this population and both conditions are highly associated with caregiver burden. We conclude that caregiver burden should be given more emphasis in palliative care managements and be factored in developing local policies.

## Data Availability

Study findings are kept at the office of Faculty of Medicine, Universiti Teknologi MARA (UiTM) Selayang campus, Jalan Prima Selayang 7, 68100 Batu Caves, Selangor, Malaysia. Data may be shared upon request of reviewers and it is subjected to the data protection regulations.

## Abbreviations

IC: Informal caregiver
PCU: Palliative Care Unit
ZBI: Zarit Burden Interview
DASS: Depression, anxiety, stress scale
